# The mediating role of resilience in the relationship between meaning in life and attitude toward death among ICU nurses: a cross-sectional study

**DOI:** 10.3389/fpsyg.2024.1414989

**Published:** 2024-11-04

**Authors:** Lifang Wang, Sisi Li, Xiaorong Liu, Rong Li, Ran Li

**Affiliations:** School of Nursing, Guangzhou Medical University, Guangzhou, Guangdong, China

**Keywords:** intensive care unit nurses, attitude toward death, meaning in life, resilience, structural equation modeling

## Abstract

**Background:**

The majority of elderly individuals prefer to spend their final days in the Intensive Care Unit (ICU). ICU nurses are key providers in hospice care, and their attitudes toward death influence the quality of end-of-life nursing. Positive psychology emphasizes promoting positive attitudes toward death. A sense of meaning in life and resilience are critical aspects of positive psychology, which are essential for shaping ICU nurses’ attitudes toward death.

**Objective:**

This study aims to investigate whether a sense of meaning in life indirectly affects attitudes toward death through the mediation of resilience.

**Method:**

A total of 212 ICU nurses from four tertiary general hospitals in Guangdong and Fujian provinces, China, participated in this study. They completed an online questionnaire, including the Death Attitude Profile-Revised, the Connor-Davidson Resilience Scale, and the China Meaning in Life Questionnaire for ICU nurses.

**Results:**

ICU nurses scored highest in natural acceptance (3.26 ± 0.53), followed by escaping acceptance (2.54 ± 0.59), and fear of death (2.53 ± 0.78). The proposed model fit the data well. Both the presence of meaning and the search for meaning were positive predictors of resilience (*β* = 0.313, *p* < 0.05; *β* = 0.256, *p* < 0.05). Resilience, the presence of meaning, and the search for meaning positively predicted natural acceptance (*β* = 0.299, *p* < 0.05; *β* = 0.294, *p* < 0.05; *β* = 0.177, *p* < 0.05). Conversely, these factors negatively predicted fear of death (*β* = −0.179, *p* < 0.05; *β* = −0.251, *p* < 0.05; *β* = −0.192, *p* < 0.05) and escaping acceptance (*β* = −0.208, *p* < 0.05; *β* = −0.208, *p* < 0.05; *β* = −0.219, *p* < 0.05). Mediation analysis indicated that resilience had a partial mediating role.

**Conclusion:**

The presence of meaning and the search for meaning influence attitudes toward death, with resilience serving as a partial mediator. It is recommended that nursing administrators consider ICU nurses’ attitudes toward death and emphasize the importance of meaning in life and resilience in death education. This approach can help protect the psychological well-being of ICU nurses, promote a deeper understanding of life’s meaning, and develop a scientific perspective on death. Such measures are crucial for providing better humanistic care and psychological comfort to terminally ill patients and their families, thereby improving the quality of end-of-life care.

## 1 Introduction

With advancements in medical technology, increased average life expectancy, and improved living standards, hospice care focusing on the “quality of dying” has gained attention. In 2020, 18.7% of China’s population will be elderly, and many with chronic illnesses and malignant tumors may qualify for hospice care. Hospice care services in China face significant challenges (Zhihong and Yan, 2023). Studies indicate that an increasing number of elderly people are spending their final month in the intensive care unit (ICU) (Cook and Rocker, 2014; Khandelwal and Curtis, 2014). ICU patients often have severe conditions and a higher mortality risk. Data shows that ICU patient mortality rates abroad range from 6.4 to 40.0% (Sakr et al., 2012; Brinkman et al., 2013; Mezzaroba et al., 2022), while in China, the rates vary between 12.8 and 45.6% (Pengcheng and Tian, 2023). Consequently, ICU nurses bear significant responsibility in providing end-of-life care. Beyond improving their advanced life support skills, they must also develop a proper perspective on life and death. Due to traditional Chinese culture, discussing death is often avoided. Nurses, too, may feel anxious and struggle to confront and accept death (Cardoso et al., 2021; He and Li, 2022). In 2015, the Economist Intelligence Unit (EIU) reported that the quality of life for terminally ill patients in mainland China was relatively low (He et al., 2023). Nurses, as primary hospice care providers, play a crucial role in the quality of life of these patients. Their attitudes toward death can influence their approach to hospice care and affect their nursing behavior toward terminally ill patients (Costello, 2006; Meng et al., 2019; Mastroianni et al., 2015; Zhang et al., 2019). Nurses with objective and impartial views on death and the value of life naturally understand the significance of hospice care, allowing them to meet the nursing needs of terminally ill patients and their families with a positive hospice care attitude, providing humanistic care and psychological comfort, which helps improve the quality of end-of-life care services (Meng et al., 2019). Additionally, evidence suggests that a scientific attitude toward death is essential for ICU nurses to provide effective hospice care (Cho and Kang, 2017; Liu, 2023). Therefore, strengthening the development of ICU nurses’ attitudes toward death is vital.

Attitude toward death refers to an individual’s consistent and evaluative psychological disposition toward death, encompassing five components: fear of death, death avoidance, natural acceptance, approach acceptance, and escape acceptance (Dezutter et al., 2009). Recently, nursing management has increasingly focused on nurses’ attitudes toward death due to its relation to their work competency, coping strategies regarding death, physical and mental health, and professional conduct. Research indicates that nurses who naturally accept death are more likely to demonstrate positive caring behaviors (Braun et al., 2010; Meng et al., 2019; Zhang et al., 2019). In contrast, those who avoid or fear death often face conflicts between their personal and professional roles, leading to a reduced ability to provide spiritual and psychological support to patients and their families (Peters et al., 2013; Kudubes et al., 2021). This suggests that a negative attitude toward death can adversely affect the quality of care. Furthermore, positive attitudes toward death correlate with higher levels of personal achievement and self-worth among nurses, which in turn leads to greater job satisfaction and improved care quality (Jing et al., 2012). Studies have shown that ICU nurses are frequently exposed to near-death or death events, which can lead to anxiety, loss, fear, and even post-traumatic stress disorder (PTSD). This exposure can increase the likelihood of quitting, complicating the provision of end-of-life care (Cho and Kang, 2017; Liu, 2023; Lian et al., 2022; Arli, 2023). Additionally, Braun et al. (2010) suggested that an individual’s attitude toward death is often influenced by their understanding of death. Ay and Öz (2019) found that the more nurses comprehend concepts related to death, such as hospice care, death, and euthanasia, the more positive their attitude toward death becomes. Consequently, death education has become a crucial method for promoting a correct attitude toward death (Testoni et al., 2018). With an aging population, China urgently needs to implement death education for ICU nurses and improve their training in perspectives on life and death to meet end-of-life care demands. However, death education in China began late, has an overly complex curriculum system, and lacks sufficient research on ICU nurses’ attitudes toward death and related factors. Therefore, it is necessary to explore further the influencing mechanisms of factors related to ICU nurses’ attitudes toward death to establish effective and targeted death education programs for ICU nurses.

Life ultimately culminates in death, which highlights the finite nature of existence. People increasingly recognize this and actively contemplate its purpose and significance (Testoni et al., 2018). The sense of meaning in life involves individuals’ subjective assessment of life’s goals and meaning, as well as their sense of achievement and satisfaction. This concept is divided into two parts: the presence of meaning and the search for meaning (Steger et al., 2006). Durlak (1972) suggested that life’s meaning is a vital component of mental health. Individuals with a strong sense of meaning in life can increase resilience, reduce the fear of dying, and approach it with a positive attitude. Studies have shown a significant positive relationship between meaning in life and the natural acceptance of death among nurses (Cardoso et al., 2021). The stronger it is, the more likely individuals are to view and face death rationally (Ran et al., 2018). Chinese researchers have found that frequent interactions with dying patients and their families provide oncology nurses with opportunities for self-reflection and contemplation of life’s meaning, which shifts their perspectives and attitudes toward death (Wang, 2022). Conversely, a strong sense of meaning in life negatively predicts fear of death and avoidance acceptance among nurses (Ran et al., 2018; Xiaomin et al., 2021). For nurses who may experience emotional distress, applying the meaning of life when caring for terminal patients is particularly important (Zheng et al., 2015). The evidence suggests a significant correlation between the meaning of life and attitudes toward death. Therefore, many nursing managers view the sense of meaning in life as essential for shaping attitudes toward death. However, no research has yet examined the relationship between ICU nurses’ sense of meaning in life and their attitudes toward death. Hence, the hypothesis is proposed that there is a correlation between ICU nurses’ sense of meaning in life and their attitudes toward death.

Resilience refers to an individual’s ability to adapt when facing stress and adversity (White et al., 2008). Nurse resilience is characterized as a complex, dynamic process that helps nurses actively adjust to workplace stressors, prevent psychological harm, and maintain high-quality patient care (Cooper et al., 2020). Schreiber et al. (2019) found that resilience can protect nurses caring for infected patients from various psychological and mental health issues. Edo-Gual et al. (2015) emphasized that resilience is a crucial emotional skill for coping with death. Jiao et al. (2023) reported that medical students’ resilience predicts their attitudes toward death, with those having higher resilience more likely to view death positively and face various challenges and stresses courageously. Wang (2022) showed that oncology nurses with strong resilience could maintain a healthy psychological state when dealing with dying patients and their families. These studies suggest that resilience positively influences attitudes toward death. Compared to nurses in general departments, ICU nurses experience greater pressure from end-of-life care and psychological stress throughout their careers and thus need higher resilience to develop a proper attitude toward death. Therefore, we predict that ICU nurses’ resilience will be a predictor of their attitude toward death in this study.

The sense of meaning in life and psychological resilience are key elements of positive psychology, acting as protective factors for mental health. A strong sense of meaning in life can promote psychological resilience by reducing uncertainty and related distress (Du et al., 2017; Yela et al., 2020; Ostafin and Proulx, 2020). According to the dynamic model of psychological resilience, resilience helps individuals adapt well by utilizing all protective resources (Connor and Davidson, 2003). Folkman (1997) suggested that resilience can influence the relationship between the sense of meaning in life and mental health. For example, Huayu and Guangming (2019) found that college students who actively seek meaning in life are more likely to use their psychological resilience to overcome boredom, thereby adapting better to their academic lives. Ya'nan and Xiaoyu, 2023 reported that final-year medical students who struggle to find meaning in life may remain resilient yet pessimistic, leading to anxiety and impeding their future professional growth. Han et al. (2023) demonstrated that nurses’ psychological resilience improved their post-traumatic growth (*PTG*) through a sense of meaning in life during the COVID-19 pandemic. This evidence indicates that the sense of meaning in life can promote positive psychological states and mitigate negative psychological disturbances through psychological resilience. Therefore, we anticipate that resilience will mediate the relationship between the sense of meaning in life and the attitude toward death among ICU nurses.

Based on the literature review, this study aims to examine the relationships between meaning in life, resilience, and attitudes toward death, and to determine if resilience mediates the relationship between meaning in life and attitudes toward death. Understanding these relationships is crucial for developing strategies in death education to improve ICU nurses’ attitudes toward death.

## 2 Materials and methods

### 2.1 Study design and participants

ICU nurses were selected through convenience sampling from four tertiary grade A hospitals in Fujian and Guangdong provinces, China. The inclusion criteria were: (1) ICU nurses holding a qualification certificate from the People’s Republic of China, and (2) having worked in the intensive care unit for more than 1 year and agreeing to participate. Nurses in training and those on leave were excluded. According to the Kendall sample size estimation method, the sample size is generally 5 ~ 10 times the number of items. With 20 items in this study and a 10% attrition rate, the sample size range was 110 ~ 220. Additionally, considering that structural equation modeling requires a sample size of more than 200, a total of 216 questionnaires were collected, with 212 valid responses (effective response rate: 98.15%).

### 2.2 Data collection

The study was conducted in Fujian and Guangdong Provinces, China, from July to August 2022. Prior to data collection, nursing managers from the four tertiary hospitals were contacted and briefed on the study’s objectives, requesting their assistance with the survey. An online questionnaire platform^1^ was used for data collection. The link to the online questionnaire was distributed to the WeChat group of ICU nurses working at the four hospitals. Researchers explained the study’s objectives to the participants, assured them of confidentiality, and informed them about the time required to complete the questionnaire (approximately 10 ~ 15 min). Upon agreeing to participate, informed consent was obtained, and participants completed the Meaning in Life Questionnaire, the Resilience Scale, and the Death Attitude Profile-Revised.

#### 2.2.1 General characteristics questionnaire

The general information questionnaire was designed by the researchers and included details such as gender, age, marital status, fertility, academic status, years of work, title, religious belief, whether participants had received education on death and life sensitivity, and whether the hospital intended to provide education on death and vitality.

#### 2.2.2 Meaning in life questionnaire

The Meaning in Life Questionnaire (MLQ) was created by Steger et al. (2006). This scale consists of 10 items divided into two subscales: the presence of meaning (e.g., “I understand the meaning of my life”) and the search for meaning (e.g., “I am looking for a purpose or mission in my life”), each containing 5 items. This study used the Chinese version of the *MLQ* revised by Mengcheng and Xiaoyang (2008). Items are rated on a Likert 7 scale, ranging from 1 (completely unsatisfactory) to 7 (completely satisfactory), with the second item reverse scored. The total score ranges from 10 to 70, with higher scores indicating a greater sense of meaning in life. In this study, the Cronbach’s *α* coefficients for the two subscales were 0.92 and 0.887, respectively.

#### 2.2.3 Resilience scale

The Resilience Scale, developed by Connor and Davidson (2003), includes three dimensions: tenacity (13 items), strength (8 items), and optimism (4 items). Participants rate items on a 5-point Likert scale (from 0 = “never” to 4 = “always”). The total score ranges from 0 to 100 points, with higher scores indicating greater resilience. The Chinese version of the Resilience Scale has demonstrated good reliability and has been validated by multiple studies (Meng et al., 2019; Cheng et al., 2020). In this study, the scale showed strong reliability, with a Cronbach’s *α* of 0.942.

#### 2.2.4 Death attitude profile-revised

The Death Attitude Profile-Revised was developed by Wong et al. (1994). This study utilized the Chinese version of the Death Attitude Profile-Revised (Zhu and Shi, 2011) to assess ICU nurses’ attitudes toward death. The scale comprises 25 items across 5 subscales: fear of death, death avoidance, escape acceptance, neutral acceptance, and approach acceptance. A Likert 4-point scale is used (from 1 = “strongly disagree” to 4 = “strongly agree”). Scores for each dimension are averaged by the number of items, and the scale does not calculate a total score but rather interprets attitudes based on individual dimension scores. Higher scores indicate a stronger tendency towards the attitude represented by each dimension. In this study, Cronbach’s α for the five subscales ranged from 0.765 to 0.885.

### 2.3 Statistical analysis

**Figure 1:**
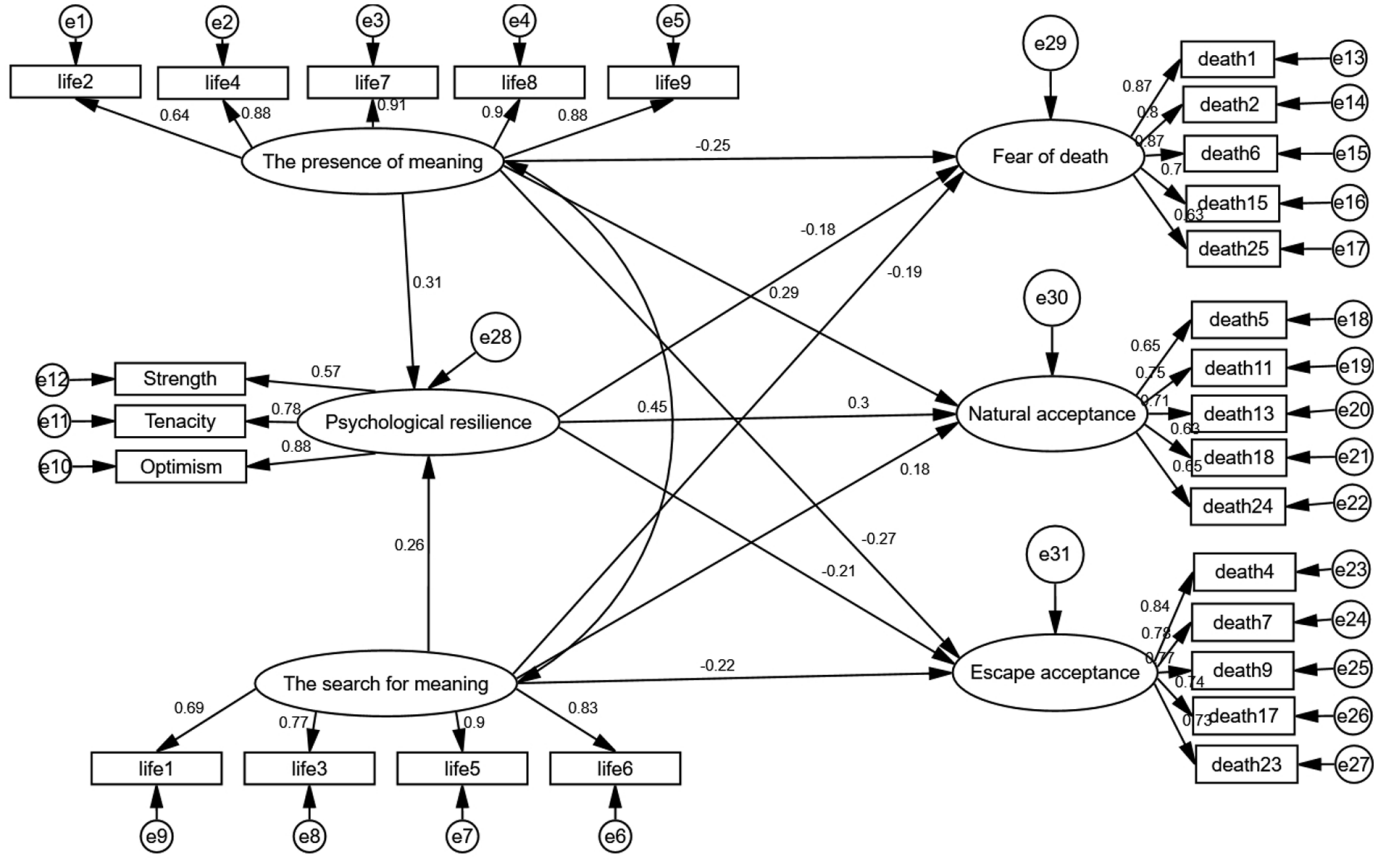
Structural equation model figure.

Statistical analysis was conducted using SPSS 25.0. The mean ± standard deviation was used to describe the levels of each variable, and Pearson’s correlation was employed to examine the relationships between variables. AMOS 26.0 was utilized to establish the structural equation model to explore the internal relationships and pathways between the sense of meaning in life, resilience, and attitude toward death. Differences were considered statistically significant at *p* < 0.05. The conceptual model is illustrated in Figure 1. The model’s fitness was evaluated using several indices: Chi-Square Minimum (CMIN), Degrees of Freedom (DF), the ratio of Chi-Square Minimum to Degrees of Freedom (CMIN/DF), Root Mean Square Error of Approximation (RMSEA), Comparative Fit Index (CFI), Incremental Fit Index (IFI), Goodness of Fit Index (GFI), and Normed Fit Index (NFI). The significance of the mediation effect was tested using Bootstrap, with a 95% confidence interval (CI) for the mediation effect calculated by sampling 2,000 times from the original data through repeated random sampling.

## 3 Results

### 3.1 General characteristics of the participants

In the final valid sample, women constituted 90.1%, while men made up 9.9%. Regarding age distribution, the sample primarily consisted of ICU nurses over 25 years old. In this study, 66% of the ICU nurses had a bachelor’s or graduate degree, 73.5% were married, 64.6% had children, 37.2% held positions as supervisor nurses or higher, and 43.9% had more than 10 years of work experience. Additionally, 21.2% of ICU nurses reported having religious beliefs. While 81.1% of ICU nurses expressed a desire for hospitals to offer education on death and the meaning of life, only 31.1% had actually received such education (Table 1).

**Table 1:** General characteristics of ICU nurses (*n* = 212).

Variable		*N*	%
Age	Under 25 years old	16	7.5
	25–35 years old	136	64.2
	Above 35 years old	60	28.3
Gender	Male	21	9.9
	Female	191	90.1
Marital status	Unmarried	56	26.4
	Married	154	72.6
	Divorced	2	0.9
Fertility situation	Yes	137	64.6
	No	75	35.4
Educational background	Technical secondary school	3	1.4
	College degree	69	32.5
	Bachelor degree	138	65.1
	Graduate degree	2	0.9
Length of nursing work	1–5 years	57	26.9
	6–10 years	62	29.2
	11–15 years	60	28.3
	16-20 years	22	10.4
	Greater than or equal to 21 years	11	5.2
Nurse title	Nurse	25	11.8
	Nurse practitioner	108	50.9
	Supervisor nurse	69	32.5
	Associate chief nurse and higher	10	4.7
Religious belief	Yes	45	21.2
	No	167	78.8
Whether education on death and the meaning of life has been conducted	Yes	66	31.1
	No	146	68.9
Whether they want hospitals to teach	Yes	172	81.1
about death and the meaning of life	No	40	18.9

### 3.2 Descriptive statistics and correlation analysis

Table 2 presents the averages, standard deviations, and correlation coefficients for each variable in this study. Comparing the scores of the DAP-R five dimensions revealed that ICU nurses’ attitudes toward death were most aligned with natural acceptance (3.26 ± 0.53), followed by avoidance acceptance (2.54 ± 0.59), and fear of death (2.53 ± 0.78). Correlation analysis results indicated that the meaning of life (both presence of meaning and search for meaning) and psychological resilience were positively correlated with natural acceptance. Conversely, the meaning of life (presence of meaning and search for meaning) and psychological resilience were negatively correlated with fear of death and escape acceptance.

**Table 2:** Descriptive statistic and variable correlations (*n* = 212).

	**1(r)**	**2(r)**	**3(r)**	**4(r)**	**5(r)**	**M ± SD (Item Score)**
1 Meaning in life	1	------	------	------	------	4.71 ± 1.09
2 The presence of meaning	0.859**	1	------	------	------	4.81 ± 1.32
3 The search for meaning	0.778**	0.347**	1	------	------	4.59 ± 1.34
4 Psychological resilience	0.445**	0.378**	0.352**	1	------	2.22 ± 0.59
5 Fear of death	−0.424**	−0.395**	−0.291**	−0.305**	1	2.53 ± 0.78
6 Death avoidance	−0.030	−0.065	0.024	0.002	------	2.54 ± 0.59
7 Natural acceptance	0.486**	0.428**	0.364**	0.394**	------	3.26 ± 0.53
8 Approach acceptance	0.003	−0.007	0.070	−0.012	------	2.32 ± 0.63
9 Escape acceptance	−0.474**	−0.410**	−0.366**	−0.346**	------	2.48 ± 0.69

**p < 0.01.

### 3.3 Mediating effect analysis

A structural equation model was constructed based on literature reviews and correlation analysis to examine the mediating role of psychological resilience in the relationship between the meaning of life (both presence of meaning and search for meaning) and attitudes toward death (natural acceptance, fear of death, and escape acceptance). The model results (Figure 1) indicate that psychological resilience did not significantly mediate the relationship between the meaning of life, death avoidance, and approach acceptance; only results for natural acceptance, fear of death, and escape acceptance were reported. The model showed a good fit with the data (CMIN/DF = 1.445, GFI = 0.867, RMSEA = 0.046, CFI = 0.957, NFI = 0.875, and IFI = 0.958), indicating a satisfactory fit (Table 3). The model revealed positive predictive relationships between meaning in life (presence of meaning and search for meaning) and psychological resilience (*β* = 0.313, *p* < 0.01; *β* = 0.256, *p* < 0.05); between meaning in life (presence of meaning and search for meaning) and psychological resilience and natural acceptance (*β* = 0.299, *p* < 0.05; *β* = 0.294, *p* < 0.05; *β* = 0.177, *p* < 0.05); and negative predictive relationships between meaning in life (presence of meaning and search for meaning) and psychological resilience and death avoidance (*β* = −0.179, *p* < 0.05; *β* = −0.251, *p* < 0.05; *β* = −0.192, *p* < 0.05) and approach acceptance (*β* = −0.208, *p* < 0.05; *β* = −0.208, *p* < 0.05; *β* = −0.219, *p* < 0.05) (Table 4).

**Table 3:** Model fit indices.

**Indicator**	**CMIN**	**DF**	**CMIN/DF**	**GFI**	**RMSEA**	**CFI**	**NFI**	**IFI**
Ideal value	–	–	<3	>0.9	<0.08	>0.9	>0.9	>0.9
Acceptable value	–	–	<5	>0.8	<0.10	>0.8	>0.8	>0.8
Fitted value	450.968	312	1.445	0.867	0.046	0.957	0.875	0.958

**Table 4:** Summary of model coefficients.

**Independent variable**	**Dependent variable**	**Unstandardized Path coefficient**	**Standardized path coefficient**	**Standard error**	***z* (C.R.)**	***p***
The presence of meaning	Psychological resilience	0.176	0.313	0.047	3.722	***
The search for meaning	Psychological resilience	0.152	0.256	0.05	3.065	0.002
Psychological resilience	Fear of death	−0.163	−0.179	0.077	−2.11	0.035
Psychological resilience	Natural acceptance	0.231	0.299	0.068	3.388	***
Psychological resilience	Escape acceptance	−0.217	−0.208	0.086	−2.506	0.012
The presence of meaning	Fear of death	−0.129	−0.251	0.044	−2.925	0.003
The presence of meaning	Natural acceptance	0.127	0.294	0.038	3.352	***
The presence of meaning	Escape acceptance	−0.16	−0.273	0.049	−3.259	0.001
The search for meaning	Fear of death	−0.104	−0.192	0.045	−2.297	0.022
The search for meaning	Natural acceptance	0.081	0.177	0.038	2.126	0.034
The search for meaning	Escape acceptance	−0.135	−0.219	0.051	−2.669	0.008

***p < 0.001.

The Bootstrap method was used to estimate confidence intervals. By repeating 2,000 samples and calculating 95% confidence intervals, the results showed that the six paths’ 95% confidence intervals did not include 0, indicating statistical significance. This confirmed that resilience partially mediated the relationship between the meaning of life (presence of meaning and search for meaning) and attitudes toward death (natural acceptance, fear of death, and escape acceptance). Detailed test results are presented in Table 5.

**Table 5:** Results of mediation effect tests.

**Path relationship**	**Direct effect**	**Indirect effect**	**Bias-corrected(95%)**		***p***	**Conclusion**
			**Lower Bounds**	**Upper Bounds**		
The presence of meaning → Psychological resilience → Fear of death	−0.129(0.003)	−0.029	−0.089	−0.003	0.025	Partial mediation
The presence of meaning → Psychological resilience → Natural acceptance	0.127(***)	0.04	0.014	0.101	0.001	Partial mediation
The presence of meaning → Psychological resilience → Escape acceptance	−0.160(0.001)	−0.038	−0.104	−0.007	0.011	Partial mediation
The search for meaning → Psychological resilience → Fear of death	−0.104(0.022)	−0.025	−0.089	−0.001	0.044	Partial mediation
The search for meaning → Psychological resilience → Natural acceptance	0.081(0.034)	0.035	0.006	0.097	0.009	Partial mediation
The search for meaning → Psychological resilience → Escape acceptance	−0.135(0.008)	−0.033	−0.107	−0.002	0.027	Partial mediation

***p < 0.001.

## 4 Discussion

This study investigated the impact of the presence of meaning and the search for meaning on natural acceptance, escape acceptance, and fear of death, respectively, while examining the partial mediating role of resilience. The findings are valuable for nursing managers to improve and expand the death education curriculum, aiding ICU nurses in better understanding and dealing with death.

The study compared the average scores across the five dimensions of death attitude and found that ICU nurses mainly displayed natural acceptance (3.26 ± 0.53), escape acceptance (2.54 ± 0.59), and fear of death (2.53 ± 0.78). ICU nurses tend to exhibit natural acceptance, viewing death as an inevitable part of life without fearing or welcoming it. This perspective may stem from their medical background and frequent encounters with death, allowing for a more logical view of life and death (Cardoso et al., 2021). Additionally, ICU nurses scored relatively high on escape acceptance. Zhu and Shi (2010) indicated that physical health often prompts deep reflections on death and life’s value. When experiencing extreme pain without a cure, individuals might see death as the final escape from suffering (Jinling et al., 2020). Therefore, it is speculated that ICU nurses’ tendency to escape acceptance of death might relate to the critical condition and poor prognosis of ICU patients. Furthermore, ICU nurses also experience a certain level of fear of death. According to Terror Management Theory, fear of death is a fundamental human fear that directly influences attitudes toward death (Lieberman, 2004; Yu et al., 2022). Thus, the fear of death among ICU nurses may be an instinctive response to facing death. Overall, ICU nurses’ attitudes toward death are characterized by natural acceptance, escape acceptance, and fear of death, reflecting various cognitive and emotional responses to death. Research indicates a significant correlation between nurses’ attitudes toward death and their perspectives on hospice care (Zhaoxia et al., 2014; Mastroianni et al., 2015; Huiying and Liqin, 2018). During the pandemic, Cardoso et al. (2021) found that varying levels of knowledge and skills among nurses can lead to differing attitudes toward death and different approaches to handling death-related events. Samson and Shvartzman (2018) reported that a positive attitude towards death can improve the care of dying patients. A positive death attitude, particularly one based on natural acceptance, is crucial in the hospice care process and is a key objective for nursing managers through death education (Cardoso et al., 2021; He et al., 2023). However, due to the relatively late development of China’s death education system and the influence of traditional culture, death education in China is still evolving. It currently features limited content and monotonous implementation methods, which somewhat hinder the advancement of death education. Additionally, death education is essential for providing hospice care. Healthcare providers, especially nurses, must first undergo death education to understand the nature of death and acquire coping skills. This education enables them to effectively assist and guide terminal patients and their families in facing death with courage, provide psychological counseling, ensure patients pass away peacefully with minimal suffering and no regrets, and offer support and understanding to their families. Therefore, it is recommended that nursing administrators improve the content and delivery methods of death education for ICU nurses. This will help nurses fully grasp the meaning of life, accept death as an inevitable part of life, confront and address death with bravery, and ultimately deliver high-quality hospice care services.

Consistent with a previous study by Ran et al. (2018) and Cardoso et al. (2021), this study found that the presence of meaning and the search for meaning were positively correlated with natural acceptance and negatively correlated with fear of death and escape acceptance. Barnett et al. (2019) observed that nurses with a higher sense of meaning may develop greater self-esteem, which helps them resist the negative effects of death. Humans are inherently driven to create and seek meaning, and pursuing meaning in life is one of the best ways to alleviate the fear of death (Zhang et al., 2019). Death challenges the possibility of achieving fundamental life goals and beliefs in life’s meaning (Barnett et al., 2018). After traumatic events involving the threat of death, individuals often re-evaluate the events and construct new meanings (Park and George, 2013). Some studies suggest that a lack of meaning in life can lead to feelings of boredom, depression, despair, and a loss of the will to live, making death seem like a form of liberation, and thus resulting in escaping acceptance (Pinquart, 2002; Xiaomin et al., 2021). Therefore, enhancing the sense of meaning in life is crucial for cultivating a scientific view of life and death. Amenta (1984) stated that the sense of meaning in life is an important and effective indicator of death education. A Chinese Taiwanese scholar noted that the essence of death education lies in exploring the nature of death, encouraging individuals to deeply consider their relationships with others, society, nature, and various topics related to dying and mourning. This process helps people understand the true meaning and value of existence, thereby overcoming fear and anxiety about death (Zhang et al., 2019). Nursing administrators should emphasize the sense of meaning in life as a critical component of death education for ICU nurses. This approach can help ICU nurses gain a deeper understanding of life’s meaning, actively contemplate and pursue the significance and value of existence, and eliminate negative psychological attitudes like fear and avoidance of death. By promoting an objective, calm, and accepting mindset towards death, ICU nurses can provide comfort, care, and patience to patients and their families, creating a comfortable medical environment, offering warm emotional support, and providing necessary psychological comfort.

The key finding of this study is that ICU nurses’ resilience is positively correlated with natural acceptance and negatively correlated with fear of death and escape acceptance. This suggests that ICU nurses with resilience experience fewer negative psychological processes when dealing with death and are more likely to naturally accept death and life’s finite nature. Wang (2022) found that oncology nurses with strong psychological resilience can maintain a positive mindset when facing death and have confidence in providing end-of-life care. Similarly, numerous studies have shown that psychological resilience can buffer the negative psychological impacts of stressful events, such as adversity, tragedy, and trauma experienced by nurses in clinical work, effectively maintaining their physical and mental balance and promoting a positive state of development and adaptation (Luthar et al., 2000; Kalisch et al., 2017; Cooper et al., 2020). Therefore, it is essential to recognize the role of resilience in ICU nurses’ psychological state when confronting near-death or death situations.

Another important finding is that ICU nurses’ resilience partially mediated the relationship between the sense of meaning in life (presence of meaning and the search for meaning) and attitudes toward death (natural acceptance, escape acceptance, and fear of death).This indicates that ICU nurses’ attitudes toward death can be predicted through their resilience. Kalisch et al. (2017) suggest that a sense of meaning in life can enhance resilience and improve psychological adaptation, fulfilling the psychosocial needs arising from stressful events. During the COVID-19 pandemic, some scholars showed that searching for meaning and value in life can provide people with positive psychological qualities and goal-oriented motivation when coping with stressors, rather than avoiding them (Ostafin and Proulx, 2020; Yildirim et al., 2021). Therefore, it is suggested that ICU nurses with a strong sense of meaning in life can empower themselves with positive energy and support, thus exhibiting high resilience when facing death events. Resilience is regarded as a dynamic process that can mediate between a sense of meaning in life and mental health (Folkman, 1997; Ya'nan and Xiaoyu, 2023). The findings of this study align with previous research. For example, psychological resilience was found to mediate the relationship between a sense of meaning in life and academic boredom among Chinese college students (Huayu and Guangming, 2019). Additionally, resilience was shown to mediate the relationship between a sense of meaning in life and anxiety or depressive emotions among fourth-year medical students (Ya'nan and Xiaoyu, 2023). Korean researchers discovered that resilience can improve the relationship between nurses’ sense of meaning in life and post-traumatic growth (Han et al., 2023). Thus, it is essential to enhance ICU nurses’ resilience. Southwick et al. (2014) suggested that psychological resilience is not an innate trait but can be learned or developed through various coping strategies tailored to individual conditions. For instance, Foureur et al. (2013) utilized mindfulness-based stress reduction therapy to help nursing staff better manage work-related stress and improve physical and mental health. Increased resilience levels were also observed through mindfulness-based meditation training. Mealer et al. (2014) conducted a 12-week multimodal resilience training program for 27 ICU nurses, which not only boosted individual resilience levels but also reduced anxiety, depression, and burnout among the nurses. Chesak et al. (2015) implemented the Stress Management and Resilience Training (SMART) curriculum with newly employed nurses, resulting in higher resilience levels in the experimental group post-training. These methods involved consciously guiding nurses to adjust their cognitive responses, adapt to stress, and increase their adaptability to internal and external environments, supporting constructive problem-solving and promoting effective outcomes. Therefore, it is recommended to develop multimodal resilience training programs within ICU nurses’ death education.

Our findings provide a theoretical foundation for death education among ICU nurses. Nursing administrators can use these results to develop advanced courses in death education and establish clearer educational objectives to scientifically improve ICU nurses’ attitudes toward death. Additionally, the effectiveness of death education can be assessed through measures of meaning in life and psychological resilience. It is essential to create specific death education courses and fully utilize internal hospital resources for ICU nurses, helping them develop psychological resilience and a sense of meaning in life. This approach encourages nurses to view death as a natural part of life without fear or anxiety, allowing them to face and accept it calmly, maintain hope, and employ reasonable strategies to manage the consequences of death.

## 5 Limitations

This cross-sectional survey, which explores mediating effects using structural equation modeling, has some limitations. ICU nurses were recruited through convenience sampling from four tertiary hospitals in Fujian and Guangdong provinces, China. These samples may not represent all ICU nurses in mainland China, limiting the generalizability of the results. Future research should expand the sample size or divide it into different groups, and conduct multi-center surveys and longitudinal studies. Additionally, the use of an online self-assessment scale to collect data may introduce reporting bias.

## 6 Conclusion

This study examined the structural relationships among ICU nurses’ sense of meaning in life, resilience, and attitudes toward death. The results confirmed the correlations among these variables and the partial mediating role of resilience. We suggest that ICU nurses’ attitudes toward death should receive greater attention as an important professional quality. By establishing multi-level death education courses, we can improve the sense of meaning in life and resilience, enabling ICU nurses to naturally accept death and provide high-quality care for dying patients. Future research should focus on the mechanisms and pathways influencing attitudes toward death, providing a theoretical basis and empirical evidence for developing more scientific death education strategies.

## Data Availability

The original contributions presented in the study are included in the article/supplementary material, further inquiries can be directed to the corresponding author.

## Footnotes

^1^https://www.wjx.cn/
